# Cortical morphometric similarity patterns and molecular signatures across ischemic stroke recovery

**DOI:** 10.3389/fneur.2025.1644413

**Published:** 2025-11-19

**Authors:** Xiuen Chen, Jialan Liang, Chengdi Deng, Zhilin Yu, Ziming Ye, Chao Qin, Yanyan Tang

**Affiliations:** 1 Department of Neurology, The First Affiliated Hospital of Guangxi Medical University, Nanning, Guangxi Province, China; 2 Department of Radiology, The First Affiliated Hospital of Guangxi Medical University, Nanning, Guangxi Province, China

**Keywords:** ischemic stroke, morphometric similarity networks, cortical difference, gene expression, stroke recovery

## Abstract

**Background:**

Ischemic stroke is associated with widespread and dynamic brain structural alterations, but the relationship between these changes and underlying molecular signatures across different post-stroke stages remains unclear. Therefore, we examined the brain structural difference of patients with 3 T high resolution magnetic resonance, further explored special molecular signatures across ischemic stroke recovery stages.

**Methods:**

Among 170 participants were recruited, including 60 acute/subacute ischemic stroke patients, 59 chronic ischemic stroke patients, and 51 healthy controls. Morphometric similarity networks (MSNs) were constructed by calculating Pearson correlations between cortical morphometric feature vectors across 308 regions. Group differences in MSN strength were assessed using covariate-adjusted linear regression. We further applied partial least squares regression to link regional MSN differences with gene expression data from the Allen Human Brain Atlas, identifying molecular signatures across ischemic stroke recovery stages.

**Results:**

Regional MSN differences differed by stroke stage, predominantly involving frontal-temporal cortices in acute/subacute ischemic stroke and widespread cortical areas in chronic ischemic stroke. Regional MSN differences were associated with cortical transcriptional gradients, identifying key stroke-related genes (e.g., KIF5B, C4orf3, APMAP, STOML1). Functional analysis highlighted molecular signatures linked to neuronal changing, axonal transport, and protein homeostasis.

**Conclusion:**

Our findings demonstrate stage-specific morphometric differences and molecular signatures associated with different stages of ischemic stroke, highlighting regionally distinct structural and transcriptomic associations. These insights may facilitate targeted interventions aimed at improving functional outcomes across different stages of stroke.

## 1 Introduction

Ischemic stroke (IS) is a leading cause of long-term disability and mortality worldwide, with a growing burden on public health systems due to its high incidence and recurrence rate (1). Understanding the neural and molecular signatures underlying stroke-induced brain alterations across different stages of recovery remains a critical challenge in the field of neuroimaging and neurogenomics. While conventional magnetic resonance imaging (MRI) and functional MRI (fMRI) techniques have revealed considerable structural and functional alterations following stroke (2–4), these methods are often insufficient to resolve the intricate inter-regional structural differences and associated molecular mechanisms that emerge over time.

Recently, Morphometric Similarity Networks (MSNs) have emerged as a powerful framework to characterize macroscale brain architecture by integrating multiple structural features derived from high-resolution anatomical MRI (5). Unlike traditional measures that focus solely on volume or cortical thickness, MSNs offer a more comprehensive profile of cortical organization by assessing the similarity in multivariate morphometric features between brain regions. This method has shown high sensitivity in detecting subtle structural disruptions in various neuropsychiatric and neurological conditions, including schizophrenia, tremor, depressive disorder, epilepsy (6–9). However, the application of MSNs to ischemic stroke, particularly across different post-stroke stages, remains largely unexplored.

In parallel, the integration of transcriptomic data with neuroimaging phenotypes offers a novel avenue to elucidate the molecular underpinnings of brain structural changes. The Allen Human Brain Atlas (AHBA) provides spatially resolved gene expression profiles across the adult human cortex, enabling researchers to identify gene sets associated with regional brain phenotypes (10). By combining MSN alterations with postmortem gene expression data, it is possible to identify transcriptional patterns that are associated with stroke-related cortical structural differences. Partial least squares (PLS) regression has proven to be a robust multivariate approach to map the relationship between spatially distributed gene expression and imaging-derived metrics (11, 12).

Here, we present a comprehensive investigation of group-level differences in cortical morphometric similarity across individuals at different stages of IS, focusing on distinctions between the acute/subacute and chronic phases. By constructing individual MSNs based on six surface-based structural features and applying PLS regression to AHBA-derived gene expression data, we aimed to explore potential molecular correlates of stroke-related cortical network differences. Furthermore, we performed gene ontology (GO) and pathway enrichment analyses on stroke-related genes to characterize their functional roles and biological relevance. The present study integrates MSN-based cortical architecture with transcriptomic profiling in the context of IS at different clinical stages, providing novel insights into the structural and molecular correlates associated with stroke recovery.

## 2 Methods

### 2.1 Participants

Participants diagnosed with IS at the First Affiliated Hospital of Guangxi Medical University from January 2019 to March 2025 were included in the study. IS was diagnosed according to the American Heart Association and American Stroke Association criteria (13) and confirmed by MRI or brain computed tomography. Acute/sub-acute (AIS) and chronic IS (CIS) were identified according to characteristic imaging features observed across various MRI sequences (14). Exclusion criteria were as follows: (a) severe aphasia and/or impaired consciousness that compromised the ability to understand and follow instructions; (b) a history of major systemic diseases, including severe cardiac, hepatic, or renal dysfunction, autoimmune disorders, or other psychiatric illnesses; (c) coexisting severe neurological disorders, such as brain tumors or traumatic brain injury, which could potentially confound the study outcomes; (d) contraindications to MRI scanning, or poor image quality that did not meet the requirements for analysis. The healthy control (HC) group was composed of 51 individuals with comparable demographic characteristics to the patient group and had received a thorough medical examination. The study procedures received approval from the Ethics Committee of the First Affiliated Hospital of Guangxi Medical University (2025-E0302), and informed consent was obtained from each participant.

### 2.2 Imaging acquisition

Imaging data were acquired using a 3 T MRI scanner (SIEMENS, Germany). High-resolution T1-weighted anatomical images were acquired using a magnetization-prepared rapid gradient-echo (MPRAGE) sequence with the following parameters: matrix size = 256 × 256, field of view = 256 × 256 mm^2^, voxel size = 1 × 1 × 1 mm^3^, slice thickness = 1.0 mm with no inter-slice gap, echo time = 2.98 ms, repetition time = 2,300 ms, flip angle = 9°, and a total of 176 axial slices.

### 2.3 Imaging preprocessing

The three-dimensional T1-weighted images were preprocessed in surface-based space using the Computational Anatomy Toolbox (CAT12)^1^ implemented in the Statistical Parametric Mapping software (SPM12)^2^ framework. Briefly, preprocessing included skull stripping, tissue segmentation into gray matter, white matter, and cerebrospinal fluid, as well as estimation and reconstruction of the cortical surface. The cortical thickness was computed using the projection-based thickness method, which involves correction for topological defects, spherical mapping, and registration to a common surface template (15). In addition, CAT 12 was also used to estimate other morphological metrics, including fractal dimension, gyrification index, central surface, mean curvature, square root of sulcal depth, and cortical thickness (8, 16). We calculated these indices for each participant using default parameter settings. The morphometric maps were resampled to the fasverage template and smoothed using a Gaussian kernel with full width at half maximum (FWHM) after they were obtained. To be more precise, we took the cortical thickness maps for each person and made them smoother using something called a Gaussian kernel. The width of this kernel was 15 mm at full width. Other maps (such as fractal dimension maps) were made smooth using a Gaussian kernel with a FWHM of 25 mm, as explained by Ruan et al. (17).

### 2.4 Construction of MSNs

We utilized the methods of Seidlitz et al. (5) to generate MSNs with the T1-weighted restricted parameters by using CAT 12. To be more specific, the outer surfaces of the brain were divided into 308 sections, of roughly equivalent dimensions (~500 mm^2^), by using a backtracking algorithm (18). These sections were based on 68 sections of the brain as shown in the Desikan–Killiany atlas (19). This Desikan–Killiany parcellated atlas was transformed onto each participant’s surface to obtain an individual surface parcellation. Six morphometric features (as detailed in the Imaging processing section) extracted previously from the T1-weighted images were used for each region. To take into account differences in the values of the features, each MRI feature in each region of each image was z normalize. Pearson correlation coefficients were computed between the morphometric feature vectors of each pair of regions, thereby generating a 308 × 308 node morphological similarity matrix for each subject. This matrix was thresholded in order to construct a weighted bipartite graph with an arbitrary connection density. This process is also known as the construction of MSN. Regional MSN strength was defined as the average weighted correlation coefficient between a given region and all other regions, serving as an index of region-specific morphometric connectivity.

### 2.5 Case–control analysis of MSN strength in stroke subgroups

To assess group differences in MSN strength, we employed a linear regression model (LRM), with MSN strength as the dependent variable and group (AIS, CIS, or HC) as the main independent variable. Age, sex, and their interaction (age × sex) were included as covariates. This model was applied at both the global level (average MSN strength across all regions) and the regional level (MSNᵢ for each cortical region). For regional analyses, the full model was specified as: MSNᵢ = intercept + *β*₁ × age + β₂ × sex + β₃ × (age × sex) + β4 × group. Here, the group variable was dummy-coded, and β₄ represents the group effect (i.e., AIS vs. HC or CIS vs. HC). This model was independently fitted for each of the 308 cortical regions. Two-sided *t*-tests were used to assess statistical significance for group comparisons, and multiple comparisons were corrected using the Benjamini-Hochberg false discovery rate (FDR) method (*p* < 0.05). Regions showing significant differences in MSN strength were further visualized using brain surface maps. Spatial correlations between case–control *t*-values and healthy control MSN strength were assessed using spin permutation tests to account for spatial autocorrelation. The spin test is a spatial permutation method. It uses random rotations of spherical projections at the cortical surface. This method is more conservative than randomly shuffling locations. It preserves the data’s spatial symmetry and contiguity. Specifically, the initial procedure involved the generation of a null distribution through the implementation of 10,000 random spatial rotations of the cortical parcellation. Subsequently, the *p-*spin values were derived from the comparison to the null models.

To demonstrate the comparison of MSN strength between the HC group and the stroke subgroups (AIS and CIS), We used the Pearson correlation coefficient to compare MSN strength between the HC group and the stroke subgroups (AIS and CIS). The following terms were defined, similar to the descriptions by Dong D et al. (20). Decoupling: Positive correlation in the HC group and negative correlation in the stroke groups indicates that structurally similar regions become less similar. Dedifferentiation: Negative correlation in the HC group and positive correlation in the stroke groups indicates that structurally dissimilar regions become similar. Hypercoupling: Positive correlation in both the HC group and the stroke groups but stronger correlations in the stroke groups indicates that similar regions become more similar. Hyperdedifferentiation: Negative correlation in both the HC group and the stroke groups but weaker correlations in the stroke groups indicates that dissimilar regions become more dissimilar.

### 2.6 Correlation analyses between MSN features and clinical severity

To investigate the associations between MSN features and clinical severity, we conducted correlation analyses between MSN strength and clinical scores, including the National Institutes of Health Stroke Scale (NIHSS) and modified Rankin Scale (mRS), for both AIS and CIS groups. Specifically, we examined: Global-level associations, by computing Spearman’s rank correlation coefficients between mean MSN strength across all 308 cortical regions and clinical scores; Regional-level associations, by calculating Spearman correlations between MSN strength in each cortical region (defined by the D-K308 atlas) and clinical scores. For the two subgroups, AIS and CIS, all relevant analyses were conducted respectively, and the Benjamini-Hochberg FDR correction method was adopted to control multiple comparisons. The correlation results were considered statistically significant when the *p* value was less than 0.05 (the *p* value after FDR correction).

### 2.7 Regional gene expression profiling

To explore the molecular basis underlying cortical morphometric similarity, we utilized transcriptomic data from the AHBA,^3^ which provides genome-wide microarray-based gene expression profiles sampled from 3,702 anatomically localized sites across six adult postmortem donors (mean age = 42.5 ± 13.4 years; 5 males, 1 female) (10). In line with established preprocessing pipelines, we employed the Abagen toolbox^4^ to map and preprocess gene expression data with respect to the D-K308 region parcellation scheme (12, 21). The preprocessing procedure included the following steps: (a) updating probe annotations to gene-level identifiers; (b) excluding probes with expression levels below the background threshold in more than 50% of the samples; (c) retaining probes with the greatest spatial consistency for each gene; (d) assigning each sample to a cortical parcel based on a 2 mm Euclidean distance criterion; and (e) applying robust sigmoid normalization to expression values within donors. As the AHBA data comprises right hemisphere samples from merely two donors, our analysis was restricted to the left hemisphere regions (*n* = 152). Although output files retained the “DK308” label for consistency with the parcellation scheme, all subsequent analyses were strictly limited to 152 left-hemisphere regions. This preprocessing resulted in a gene expression matrix comprising 152 brain regions and 15,632 transcripts. To maintain whole-genome coverage, we did not further filter low-expression or low-variance genes; instead, robustness was ensured through subsequent bootstrapping and false discovery rate (FDR) correction.

### 2.8 Association between MSN differences and gene expression profiles in IS

To explore the molecular correlates of regional MSN differences across the course of ischemic stroke, we employed PLS regression to link spatial patterns of MSN change with transcriptomic data (22). Specifically, we extracted t-statistics representing group-level differences in MSN features between acute/subacute and chronic stroke patients across 152 cortical regions of the left hemisphere. These *t*-values served as the response variable, while gene expression levels derived from the AHBA were used as predictors. Expression data were matched to the D-K308 parcellation, restricted to the left hemisphere regions for consistency.

The number of PLS components was determined by examining the cumulative variance explained across the first 15 components. Models including 1–15 components were constructed using the plsregress function, and the percentage of variance explained in the dependent variable was recorded for each component. A null distribution was then established through 1,000 permutations, in which the dependent variable was randomly shuffled and PLS was recalculated 1,000 times. The variance explained by each component was obtained. In the original data, the first component significantly exceeded the random level (*p* = 0.001), while subsequent components contributed less than 2% incremental variance. Therefore, only the first two components were able to account for a substantial proportion of variance in the response variable and exceeded the permutation-based null distribution, which led us to focus on PLS1 and PLS2. Ultimately, the first partial least squares component (PLS1), representing a weighted combination of gene expression most strongly associated with regional MSN differences in ischemic stroke, was retained for further analysis.

In addition, we estimated the stability of each gene’s loading on PLS1 using bootstrap resampling. For each gene, a Z score was calculated as the ratio of its mean weight to the bootstrap-derived standard deviation. Although the calculation of Z scores assumes approximate normality, bootstrap resampling provides an empirical estimate of weight variability, thereby reducing over-reliance on distributional assumptions. To address the inherent sign indeterminacy of PLS, bootstrap weights were aligned to the direction of the original PLS component to ensure consistency across resampling iterations. Since the sign of PLS components is mathematically arbitrary, our interpretation focused on the relative ranking of gene weights rather than their absolute direction. Confidence intervals were not computed for ROI-level PLS scores. Instead, uncertainty was primarily quantified at the gene-weight level using bootstrap standard errors, Z scores, and FDR-corrected *p*-values. The associations between ROI-level PLS scores and MSN *t*-values were further validated using spin permutation tests to account for spatial autocorrelation.

Genes were then ranked according to their Z scores, and statistical significance was determined using FDR correction with a threshold of *p* < 0.05. Genes with PLS1 Z scores greater than 3.0 were designated as PLS1+, while those with Z scores less than −3.0 were classified as PLS1−. Only genes significantly associated with PLS1 were included in subsequent spatial expression mapping and enrichment analyses. PLS1 scores were also correlated with MSN *t*-values to validate the explanatory power of the PLS model.

### 2.9 Functional enrichment analysis of stroke-associated PLS1 genes

To explore the potential biological functions of genes associated with structural network differences in ischemic stroke, we conducted GO and pathway enrichment analyses on the ranked gene lists derived from PLS regression. Specifically, genes exhibiting statistically significant differences in bootstrap-derived Z scores for each condition (acute/subacute and chronic stroke) were analyzed separately to identify enriched biological processes and signaling pathways. Furthermore, to pinpoint genes that may play a role in both early and late stages of stroke, we performed an intersection of PLS1 genes from the two time points, followed by functional enrichment analyses on the overlapping set. All results were plotted by the Bioinformatics online platform (last accessed April 1, 2025)^5^, which provides integrated tools for GO term classification and Kyoto Encyclopedia of Genes and Genomes (KEGG) pathway enrichment (23). Statistically significant terms were determined with a threshold of *p* < 0.05.

## 3 Results

### 3.1 Demographic and clinical characteristics

A total of 170 patients with IS, including 60 in the AIS and 59 in the CIS, along with 51 HCs, were included in the final analysis. The demographic and vascular risk profiles of all participants are presented in Table 1. No statistically significant differences were observed in age (AIS: 55.98 ± 8.91 years; CIS: 56.41 ± 11.43 years; HC: 54.06 ± 8.91 years) or gender distribution across the groups (*p* > 0.05). Similarly, comparisons among groups revealed no significant differences in the prevalence of major vascular risk factors, including smoking, drinking, hypertension, diabetes mellitus, dyslipidemia, coronary heart disease, and atrial fibrillation (all *p* > 0.05). Clinical severity, as measured by the NIHSS and mRS, showed a trend toward higher scores in the AIS group compared to the CIS group, although these differences did not reach statistical significance (NIHSS: *p* = 0.053; mRS: *p* = 0.060). These findings suggest comparable baseline demographic and risk factor distributions among groups, minimizing potential confounding effects in subsequent morphometric and transcriptomic analyses.

**Table 1:** Demographic and risk characteristics.

Variables	HC (*n* = 51)	Total IS (*n* = 119)	*p*	AIS (*n* = 60)	CIS (*n* = 59)	*p*
Demographic characteristics						
Age (years)	54.06 ± 8.91	56.19 ± 10.19	0.196^a^	55.98 ± 8.91	56.41 ± 11.43	0.822^a^
Gender (M/F)	29/22	80/39	0.197^b^	42/18	38/21	0.516^b^
Risk factors						
Smoking (%)	18 (35.3)	53 (44.5)	0.263^b^	29 (48.3)	24 (40.7)	0.401^b^
Drinking (%)	20 (39.2)	50 (42.0)	0.734^b^	28 (46.7)	22 (37.3)	0.300^b^
Hypertension (%)	30 (58.8)	86 (72.3)	0.084^b^	47 (78.3)	39 (66.1)	0.136^b^
Diabetes mellitus (%)	6 (18.5)	22 (18.5)	0.279^b^	14 (23.3)	8 (13.6)	0.170^b^
Dyslipidemia (%)	11 (21.6)	37 (31.1)	0.206^b^	23 (38.3)	14 (23.7)	0.085^b^
Coronary heart disease (%)	0 (0.0)	4 (3.4)	0.185^b^	2 (3.3)	2 (3.4)	0.986^b^
Atrial fibrillation (%)	0 (0.0)	1 (0.8)	0.511^b^	1 (1.7)	0 (0.0)	0.319^b^
Clinical characteristics						
NIHSS score (P25-P75)	NA	2 (2–4)	NA	3 (2–4)	2 (1–3)	0.053^c^
mRS score (P25-P75)	NA	2 (1–2)	NA	2 (1–2)	2 (1–2)	0.060^c^

HC, Healthy controls; IS, Ischemic Stroke; AIS, Acute ischemic stroke; CIS, Chronic Ischemic Stroke; M/F, Male/female; NIHSS, NIH Stroke Scale; mRS, Modified Rankin Scale; P25–P75, 25th Percentile–75th Percentile.

^a^Unpaired *t* test.

^b^Chi-square test.

^c^Mann–Whitney *U*-test (two-sided).

### 3.2 MSN differences across stroke stages

**Figure 1:**
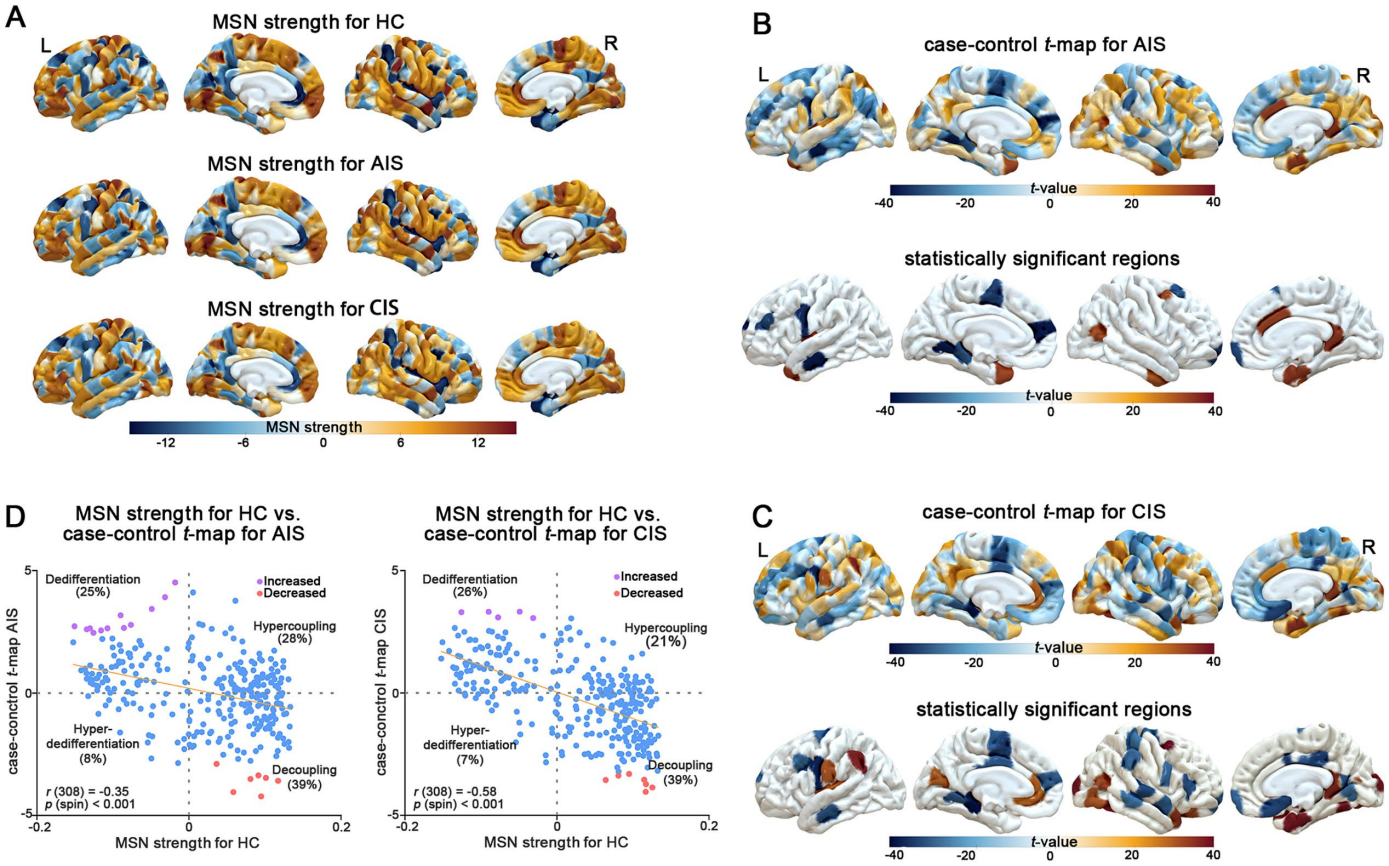
MSN differences across stroke stages. **(A)** Regional MSN strength maps for healthy controls (HC), acute/subacute stroke (AIS), and chronic stroke (CIS). **(B)** AIS vs. HC: *t*-maps (top) and significant regions (bottom) showing MSN differences. **(C)** CIS vs. HC: *t*-maps (top) and significant clusters (bottom) highlighting widespread changes. **(D)** Scatterplots of HC MSN strength vs. case–control *t*-values for AIS (left) and CIS (right), illustrating patterns of dedifferentiation, hypercoupling, hyper-dedifferentiation and decoupling.

We first constructed individual MSNs by computing interregional Pearson correlations across six cortical features derived from T1-weighted MRI data. At the group level, no statistically significant differences in global MSN strength were observed between AIS or CIS patients and healthy controls (Figure 1A). Therefore, we further examined region-specific differences in MSN to identify localized changes associated with stroke. Regional case–control *t*-maps (AIS-HC and CIS-HC) were generated by conducting linear regression analyses at each cortical region, controlling for age and sex. For each comparison, two-sided *t*-statistics were computed to quantify differences in regional MSN strength between stroke patients and healthy controls. Positive *t*-values indicate regions with increased MSN in AIS or CIS relative to controls, while negative *t*-values reflect decreased MSN. The spatial patterns of these *t*-values revealed distinct region-specific disruptions in morphometric similarity associated with the acute/subacute and chronic phases of IS. In the AIS group, significant differences in MSN strength relative to HC were predominantly observed in the frontal and temporal cortices, with additional involvement of parietal, occipital, and limbic regions (Figure 1B; Supplementary Table S1). Notably, regions showing reduced MSN strength included bilateral superior and middle frontal areas, the precentral gyrus, middle temporal cortex, and lingual gyrus. Conversely, increased MSN strength was identified in regions such as the temporal pole, insula, entorhinal cortex, cingulate cortex, and inferior parietal lobule. These findings suggest widespread and regionally divergent MSN difference during the acute/subacute phase of IS. In the CIS group, widespread differences in MSN strength were observed across multiple cortical regions compared to HC (Figure 1C; Supplementary Table S1). Reduced MSN strength was mainly observed in the bilateral prefrontal cortex, including the caudal middle frontal, rostral anterior cingulate, superior frontal, orbitofrontal, and medial prefrontal areas, as well as in the precentral gyrus, posterior cingulate cortex, parietal regions (including precuneus, supramarginal, inferior parietal), and occipital regions (including lingual and lateral occipital gyri). Increased MSN strength was primarily located in right hemisphere regions, including the caudal middle frontal, fusiform, entorhinal, insula, inferior parietal, and lateral orbitofrontal cortices, as well as the frontal pole and supramarginal gyrus. These results indicate that distinct MSN patterns are observed in the chronic phase, characterized by both enduring disruptions and regionally elevated similarity in specific cortical hubs. In both AIS and CIS groups, altered MSN connections were predominantly characterized by decoupling (39%), followed by dedifferentiation (AIS: 25%, CIS: 26%), hypercoupling (AIS: 28%, CIS: 21%), and hyperdedifferentiation (AIS: 8%, CIS: 7%) (Figure 1D). A significant negative correlation was observed between MSN strength in healthy controls and group differences (AIS: Spearman’s *r* = −0.35, *p-*spin < 0.001; CIS: Spearman’s *r* = −0.58, *p*-spin < 0.001), indicating that in the comparison between the HC group and the stroke patient group (AIS and CIS), areas with higher MSN strength in the HC group often exhibit lower MSN strength in the stroke patient group, and conversely. This negative correlation suggests that regional differences in MSN strength between the HC group and the stroke patient group are negatively correlated with MSN strength itself in the HC group. Most significantly changed connections were located in the decoupling and dedifferentiation quadrants, reflecting disrupted integration of structurally coherent regions following stroke.

### 3.3 Associations between MSN features and clinical severity

**Figure 2:**
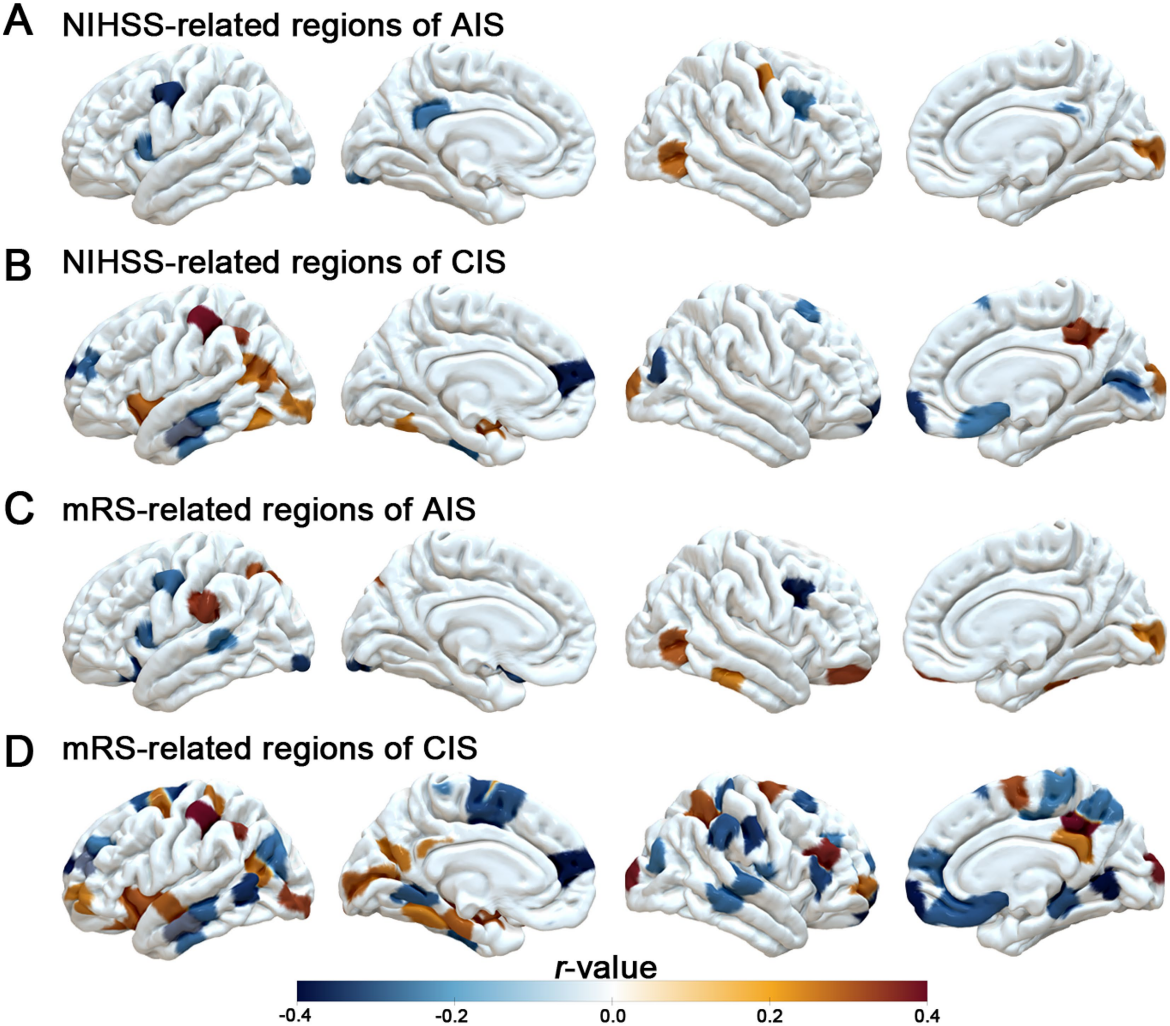
Associations between regional MSN strength and clinical scores. **(A, B)** Brain regions where MSN strength correlates with NIHSS scores in AIS and CISpatients. **(C, D)** Brain regions where MSN strength correlates with mRS scores in AIS and CIS groups. Color scale indicates correlation coefficients (*r*-values) without FDR correction, with positive and negative associations shown in orange and blue, respectively.

To explore the associations between MSN features and clinical severity, we performed Spearman correlation analyses between both global mean MSN strength and regional MSN values (based on D-K308 atlas) and clinical scores (NIHSS and mRS), separately for the AIS and CIS groups. After applying FDR correction (*p* > 0.05, Supplementary Table S2), no statistically significant correlations were observed between global mean MSN strength and either NIHSS or mRS scores in the AIS or CIS groups. At the regional level, prior to FDR correction, region-specific associations between MSN values and clinical scores were observed (Figure 2). However, after correcting for multiple comparisons, none of the associations between regional MSN values and clinical severity remained statistically significant (*p* > 0.05, Supplementary Table S3).

### 3.4 Transcriptomic correlates of MSN differences across stroke stages

**Figure 3:**
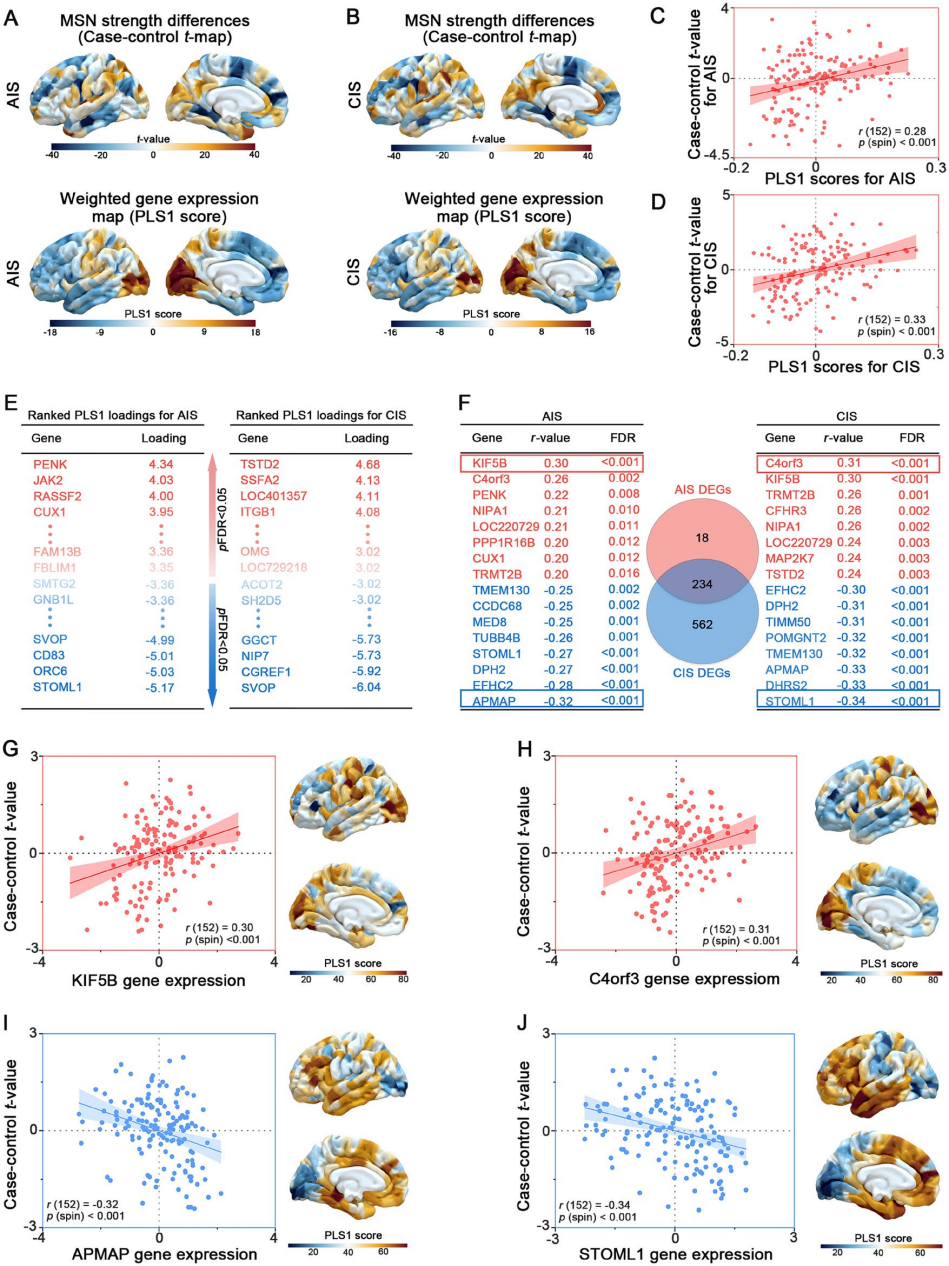
Transcriptomic correlates of MSN differences across stroke stages. **(A, B)** Case–control *t*-maps of MSN strength differences and PLS1-weighted gene expression maps in AIS and CIS groups, showing spatial correspondence. **(C, D)** Scatterplots showing significant correlations between regional PLS1 scores and MSN *t*-values in AIS (*r* = 0.33) and CIS (*r* = 0.28) groups. **(E)** Top differentially exploratory expressed genes (PLS1^+^ and PLS1^−^) identified for AIS and CIS groups based on PLS1 loading scores. **(F)** Shared and stage-specific differentially exploratory expressed genes (DEGs) associated with regional MSN differences. Left and right tables list representative DEGs in the AIS and CIS groups, respectively, ranked by Spearman *r*-values (FDR < 0.05); positively and negatively correlated genes are shown in red and blue. The central Venn diagram illustrates the number of overlapping and unique DEGs between AIS and CIS groups. **(G–J)** Examples of positively correlated genes (KIF5B, C4orf3) and negatively correlated genes (APMAP, STOML1), showing gene expression-MSN associations and spatial expression patterns aligned with *t*-map distributions.

In an exploratory PLS regression, spatial patterns of MSN disruption were associated with cortical gene expression gradients. Spatial distribution of PLS1-weighted gene expression maps found distinct anterior–posterior gradients, broadly mirroring the topography of MSN disruptions (Figures 3A,B). Furthermore, regional PLS1 scores were positively correlated with case–control *t*-values in both AIS (Spearman’s *r* = 0.33, *p*-spin < 0.001) and CIS (Spearman’s *r* = 0.28, *p*-spin < 0.001), indicating that transcriptional patterns aligned with the degree of MSN disruption across cortical regions (Figures 3C,D). These exploratory findings suggest that stroke-related MSN differences are spatially coupled with intrinsic cortical gene expression architecture. We identified differentially expressed genes based on PLS1 scores with a threshold of *p-*FDR < 0.005. In the AIS group, 37 PLS1^+^ genes (positive scores) were up regulated, while 215 PLS1^−^ genes (negative scores) were down regulated (Figure 3E). In the CIS group, a larger number of genes exhibited significant changes, with 148 PLS1^+^ genes upregulated and 648 PLS1^−^ genes downregulated (Figure 3E, Supplementary Table S4).

To further identify key genes involved in the pathogenesis of IS, we analyzed the intersection of differentially expressed genes (DEGs) between AIS and CIS. We identified 234 overlapping genes shared by AIS and CIS DEGs. Subsequently, we performed spatial correlation analysis between the expression levels of these overlapping genes and case–control *t*-values. Genes were ranked based on the Spearman’s *r*-value and statistical significance (FDR-adjusted *p* < 0.05) (Figure 3F, Supplementary Table S5). Notably, genes such as KIF5B (*r* = 0.30, *p* < 0.001), C4orf3 (Spearman’s *r* = 0.31, *p*-spin < 0.001), APMAP (Spearman’s *r* = −0.32, *p*-spin < 0.001) and STOML1 (Spearman’s *r* = −0.36, *p*-spin < 0.001) exhibited strong correlations with case–control *t*-values. The expression levels of the most highly ranked positively (or negatively) associated genes showed consistency with (or in contrast to) the distribution of variant regional changes in MSN strength (Figures 3G–J).

### 3.5 Functional enrichment of stroke-related genes

**Figure 4:**
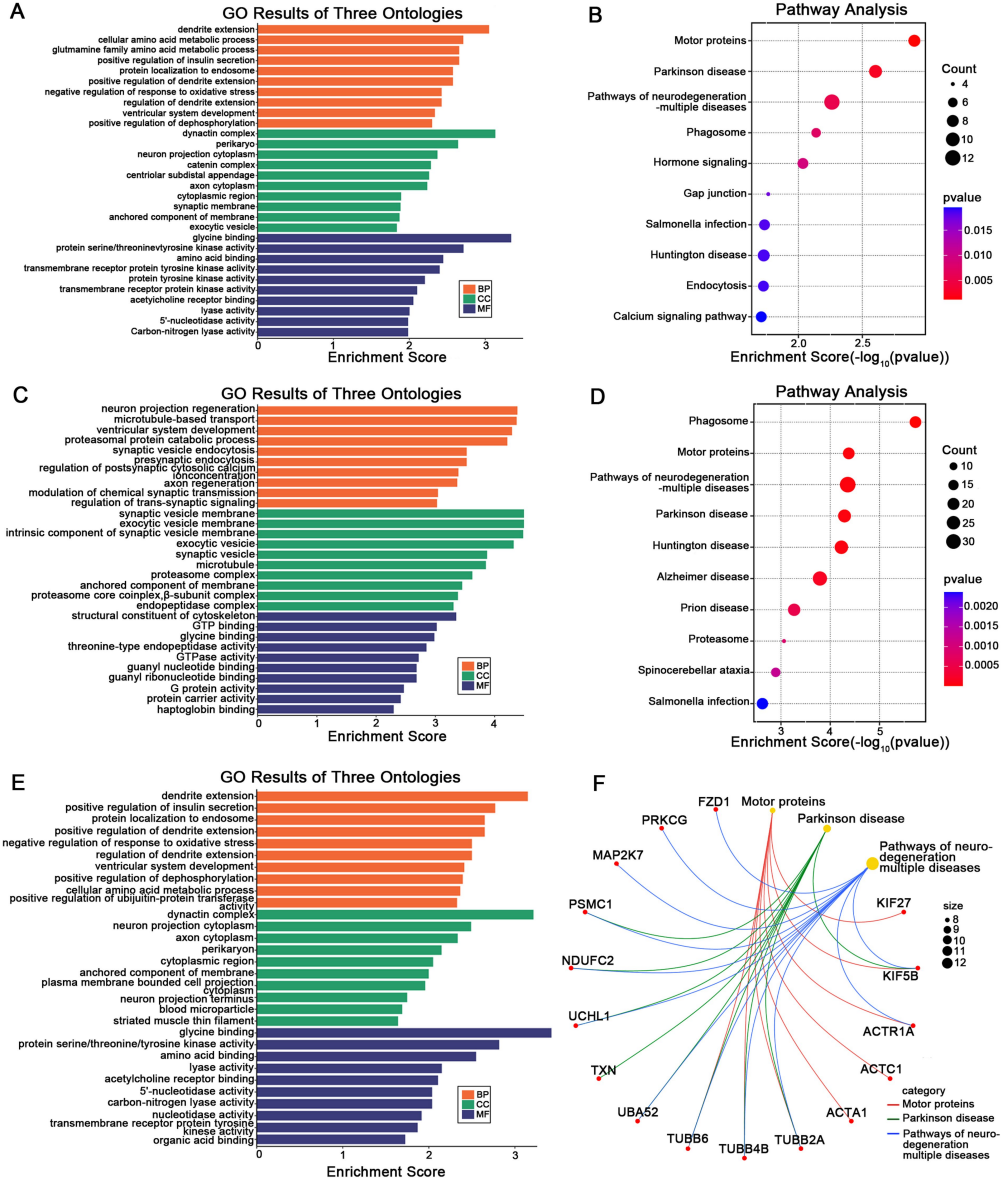
Functional enrichment of stage-specific and shared DEGs. GO **(A)** and KEGG **(B)** enrichment analysis of DEGs specifically identified in the AIS group. GO **(C)** and KEGG **(D)** enrichment analysis of DEGs specific to the CIS group. GO **(E)** and KEGG **(F)** enrichment of DEGs shared between AIS and CIS groups, reflecting common molecular signatures. In **(F)**, the gene-pathway network illustrates associations between hub genes and enriched pathways, where larger nodes represent pathway terms (scaled by –log₁₀ adjusted *p*-value) and smaller nodes represent related genes.

To elucidate the biological signatures across cortical MSN differences, GO and KEGG enrichment analyses were performed on DEGs identified in the AIS and CIS groups. In the AIS group, biological process (BP) terms were mainly enriched in dendritic extension, cellular amino acid metabolism, and insulin secretion regulation. Cellular component (CC) terms involved neuron projection cytoplasm and synaptic membranes, while molecular function (MF) terms were related to glycine binding and protein kinase activity (Figure 4A). KEGG analysis indicated significant enrichment in motor proteins, Parkinson disease, and neurodegeneration pathways (Figure 4B). In the CIS group, BP terms highlighted neuronal projection regeneration and microtubule-based transport. CC terms involved synaptic vesicle membranes and proteasome complexes, and MF terms were enriched in GTP binding and nucleotide binding (Figure 4C). KEGG analysis showed overlap with AIS, including motor proteins, phagosome, and neurodegenerative disease pathways (Figure 4D). Analysis of overlapping DEGs revealed common enrichment in dendritic extension, axon cytoplasm, and organic acid binding (BP, CC, MF respectively; Figure 4E). Shared pathways were mainly related to motor proteins, Parkinson disease, and multiple neurodegenerative disorders (Figure 4F). Gene-pathway network analysis identified hub genes bridging key biological functions. FZD1 and PRKCG were linked to motor protein regulation and neuronal signaling. KIF27, KIF5B, and ACTR1A were associated with axonal transport and cytoskeletal organization. TUBB6, TUBB4B, and TUBB2A were central to microtubule stability. PSMC1, NDUFC2, UCHL1, TXN, UBA52, and MAP2K7 were related to protein degradation and oxidative stress regulation. These hub genes are associated with cortical MSN differences in stroke and may reflect coordinated changes related to neuronal structure, intracellular transport, and protein homeostasis.

## 4 Discussion

This study compared patients in the AIS and CIS phases of ischemic stroke to investigate stage-specific differences in cortical MSN patterns. Using a data-driven, multimodal approach, we characterized the spatial distribution of morphometric changes and explored their transcriptomic associations. Although no statistically significant associations between clinical severity and MSN features were observed after multiple comparison correction, exploratory analyses revealed certain spatial trends, suggesting the presence of stage-dependent cortical structural differences during different stroke phases.

At the regional level, uncorrected correlation results showed that in the AIS group, higher NIHSS scores were associated with lower MSN values in the left frontal, cingulate, and occipital regions, as well as the right caudal middle frontal cortex; in contrast, positive correlations were observed in the right occipital and precentral regions. In the CIS group, negative correlation trends were noted in the left middle and superior frontal cortices and the right frontal pole, while positive associations were found in the bilateral inferior parietal lobules and right precuneus. Similar exploratory spatial distributions were observed for mRS scores: in the AIS group, negative trends appeared in the frontal and occipital cortices, while positive associations were found in the parietal and orbitofrontal cortices; in the CIS group, mixed positive and negative associations emerged in regions such as the middle temporal gyrus, postcentral gyrus, and superior parietal lobule. Although these results did not reach statistical significance after FDR correction, they are consistent with the concept of transneuronal diaschisis, in which focal brain injury induces remote structural and functional disturbances through mechanisms such as transneuronal degeneration, inflammatory responses, maladaptive plasticity, excitotoxicity, and necroptosis (24–30). Neuroimaging evidence shows degeneration in connected areas after stroke and severe loss of connectivity in disconnected areas (31–33). Therefore, although these trends were not statistically robust, they provided directions for hypothesis-driven research in future longitudinal or large-sample studies.

Despite the lack of statistically significant structure–function correlations between MSN and clinical severity, our transcriptomic analyses exploratory found spatial relationships between MSN patterns and cortical gene expression profiles. In both AIS and CIS groups, an anterior–posterior gradient in PLS1-weighted gene expression was observed (Figures 3A–D), indicate that intrinsic transcriptional architecture is topographically associated with regional variation in cortical MSN patterns. This finding aligns with prior evidence indicating that brain-wide morphometric and functional networks are constrained by spatial gene expression gradients (34, 35). The positive correlations between PLS1 scores and case–control *t*-values in both AIS and CIS groups underscore that regions exhibiting greater morphometric disruption also exhibit distinct transcriptomic signatures. Notably, a larger number of DEGs were detected in the CIS group, suggesting more extensive transcriptional reprogramming in the chronic phase, potentially reflecting long-term neurological changes (36) and inflammatory responses. Fury et al. used a mouse model of transient middle cerebral artery occlusion. They performed transcriptomic profiling at 10 time points after stroke. Immune responses were found to be especially affected. Immune cells persisted in distant brain regions for up 2 months (37).

Shared DEGs between AIS and CIS, including genes such as KIF5B, C4orf3, APMAP, and STOML1 (Figures 3E–J), may reflect common molecular signatures associated with stroke across different clinical stages. KIF5B, a kinase motor protein, facilitates the transport of mitochondrial and synaptic cargo and is critical for synaptic plasticity, axonal integrity and spine stability (38–41). This gene may be associated with stroke-related cortical structural variation, potentially linked to molecular pathways involving intracellular transport and neuroplasticity. C4orf3, also known as ALN, modulates ER-mediated Ca^2+^ cycling and thermogenesis, and is enriched in immune-related macrophage populations (42, 43). Given its known roles in energy balance and inflammation, this gene may be associated with molecular features relevant to metabolic and immune processes in stroke. Among the negatively correlated genes, APMAP and STOML1 showed notable associations with MSN disruption. APMAP encodes a membrane-associated protein that negatively regulates amyloid-*β* production by modulating *γ*-secretase-APP interactions and autophagy-lysosome pathways (44, 45). Its down regulation post-stroke may reflect impaired protein homeostasis and contribute to maladaptive neurodegenerative-like processes. STOML1, a protein expressed in sensory neurons, modulates ion channels and is linked to brain morphology (46–48). Its down regulation post-stroke may disrupt ion homeostasis and contribute to network vulnerability in structurally affected cortical regions.

In line with these transcriptomic associations, functional enrichment analysis of stage-specific and overlapping DEGs identified the biological pathways potentially associated with cortical MSN differences observed across stroke stages (Figure 4). In the AIS group, early transcriptional responses were enriched in dendritic extension, synaptic function, and insulin signaling regulation, suggesting acute-phase adaptations aimed at restoring neuronal communication and metabolic stability. By contrast, the CIS group demonstrated enrichment in pathways related to microtubule transport and synaptic vesicle cycling, reflecting chronic-stage demands on intracellular trafficking and synaptic maintenance. Notably, overlapping DEGs between AIS and CIS showed enrichment in pathways related to motor protein regulation and neurodegenerative disease pathways, suggesting common molecular features associated with neuronal structure and function across stroke stages. This convergence supports the notion that ischemic stroke may initiate long-lasting, neurodegeneration-like processes involving disrupted cytoskeletal dynamics, impaired proteostasis, and oxidative stress (49–51). Axonal transport within neurons is the process by which transport proteins transport corresponding proteins and other substances to axon terminals through the cytoskeleton. Defects in axonal transport components have been demonstrated to be associated with a wide range of neurological diseases, especially in the context of neurodevelopmental and neurodegenerative diseases. The restoration of axonal transport has been regarded as a potential therapeutic approach to decelerate the progression of neurodegenerative diseases for an extended period (52). The impact of ischaemic stroke on cerebral white matter is characterized by defects in axonal function, thus, protecting axonal function is of great significance for the recovery of ischemic stroke (53). Protein homeostasis is the balance of protein synthesis, folding, repair and degradation in cells. Disruptions cause diseases, including neurodegeneration. In the context of ischaemic stroke, the maintenance of protein homeostasis is pivotal for cell proliferation and functional recovery (54). Hub genes such as KIF5B, TUBB4B and UCHL1 were central to these networks, reflecting their respective roles in neuronal signaling, cytoskeletal organization and protein homeostasis (40, 41, 55, 56). Importantly, identifying molecular signatures shared across stroke stages highlights potential targets for future investigation into biological processes relevant to brain adaptation and long-term outcomes.

In summary, although this study did not reveal statistically significant structure–function relationships between MSN features and clinical scores, the combination of exploratory morphometric trends and consistent molecular signatures provided new insights into the spatial and biological signatures of post-stroke cortical deferences. These findings suggest that integrating neuroimaging with transcriptomic data may help elucidate regional structural vulnerability and potential therapeutic targets in stroke recovery. However, several limitations should be noted. First, the cross-sectional design limits causal inference and the ability to capture intra-individual dynamic changes, making it impossible to directly observe alterations in brain structure and molecular mechanisms at the individual level. Second, the gene expression data were derived from the Allen Human Brain Atlas, which is based on postmortem samples from non-stroke individuals and may therefore not fully reflect stroke-specific transcriptomic features. Third, the retrospective nature of clinical data collection precluded control of key confounders, such as lesion volume, lesion location, and time since stroke onset, all of which may significantly influence imaging and clinical outcomes. Fourth, methodological considerations related to partial least squares (PLS) analysis also warrant attention. The same dataset was used for component selection, model fitting, and gene weight inference, which could introduce a risk of overfitting. Although we employed permutation testing to establish a null distribution for component selection, bootstrap resampling to estimate the stability of gene weights, and spin permutation tests to account for spatial autocorrelation, these steps cannot fully substitute for cross-validation or external replication. In addition, the calculation of bootstrap-based Z scores assumes approximate normality, which may not hold for all genes. To address the arbitrary sign indeterminacy inherent in PLS, we aligned bootstrap weights with the direction of the original components to ensure stability across iterations, but we avoided making strong directional biological interpretations. Finally, confidence intervals for ROI-level PLS scores were not computed, and uncertainty was primarily quantified at the gene-weight level. These limitations have been described in detail in the Methods section, and the PLS results are explicitly labeled as exploratory.

To confirm the robustness and generalizability of these findings, future prospective studies should implement formal cross-validation and include independent or longitudinal cohorts, incorporate more individual-level transcriptomic data, improve measurement of unmeasured factors, and include relevant covariates. Such efforts will better support elucidating the structural-molecular interactions underlying cortical reorganization after stroke.

## 5 Conclusion

In summary, our study sheds new light on stage-specific differences in cortical morphometric similarity and its exploratory molecular correlates in ischemic stroke, highlighting the intricate spatial relationships between structural features and gene expression profiles. These findings offer insights into the structural and molecular characteristics of stroke at different clinical stages, which may inform future investigations into neurobiological processes relevant to recovery.

## Data Availability

The original contributions presented in the study are included in the article/Supplementary material, further inquiries can be directed to the corresponding authors.

## Footnotes

^1^ http://www.neuro.uni-jena.de/cat/

^2^ http://www.fil.ion.ucl.ac.uk/spm/softwarespm12

^3^ http://human.brain-map.org

^4^ https://github.com/rmarkello/abagen

^5^ https://www.bioinformatics.com.cn
